# ID 317 - Monitoramento da Segurança da Vacina Dengue Atenuada Incorporada no Brasil, 2024

**DOI:** 10.5327/2237-9622.2025.v34s1.317

**Published:** 2025-11-25

**Authors:** Cibelle Mendes Cabral, Carla Dinamerica Kobayashi, Cibelle Mendes Cabral, Martha Elizabeth Brasil da Nóbrega, Monica Brauner de Moraes, Paulo Henrique Santos Andrade, Jadher Percio

**Keywords:** efeitos colaterais e reações adversas relacionados a medicamentos, vacinação, dengue, segurança, anafilaxia, farmacovigilância

## Abstract

**Introdução:**

A vacinação é uma das intervenções mais eficazes na prevenção de doenças infecciosas, contribuindo significativamente para a redução da morbidade e da mortalidade em diversas populações. Entretanto, como qualquer intervenção, as vacinas podem estar relacionadas a eventos supostamente atribuíveis à vacinação ou imunização (Esavi). Esses eventos ocorrem após a vacinação e, embora nem sempre tenham uma relação causal com a vacina, requerem monitoramento rigoroso, para assegurar a segurança do imunobiológico em uso. Nesse contexto, o objetivo deste trabalho foi descrever os dados de Esavi para a vacina Dengue Atenuada.

**Método:**

Foram analisadas as notificações de Esavi no e-SUS Notifica entre a Semana Epidemiológica 9 de 2023 e 32 de 2024. Os Esavi de interesse e os eventos adversos de Interesse Especial (EAIE) foram identificados por meio da busca por termos de interesse no banco de dados e pela busca algorítmica utilizando . Os eventos buscados foram: anafilaxia; citopenia hematopoiética; anemia aguda; encefalite não infecciosa; meningite asséptica; hepatite aguda ou hepatite não infecciosa; insuficiência renal aguda; miopericardite não infecciosa; síndrome de Guillain-Barré; paralisia de Bell; polineuropatia inflamatória e dengue grave. Os casos identificados foram classificados conforme as definições da e da . Para o cálculo das taxas e dos coeficientes, utilizaram-se as doses administradas (DA) presentes na Rede Nacional de Dados em Saúde.

**Resultados:**

No período avaliado, foram administradas 2.911.003 doses (primeira dose: 2.381.483 [81,8%]; e segunda dose: 529.520 [18,3%]); e registradas 2.748 (94,4 por 100 mil DA) notificações para a vacina dengue atenuada. Dessas, 2.049 (74,6%; 70,4 notificações por 100 mil DA) corresponderam a Esavi e 699 (25,4%; 24,0 notificações por 100 mil DA) foram erros de imunização. Entre os Esavi, 216 casos (7,9%; 7,4 notificações por 100 mil DA) foram classificados como eventos graves. Um dos casos evoluiu para óbito, sendo posteriormente avaliado como um evento coincidente, atribuído a uma condição subjacente (fibrose cística grave). Entre os EAIE monitorados, apenas "anafilaxia" foi identificado como um sinal de segurança, com 79 casos confirmados (27,1 casos por milhão DA) e nenhum óbito. Desses, 44 (55,7%) eram do sexo feminino, com idade mediana de 11 anos (Q1: 10 e Q3: 13). Apenas 5 (6,3%) casos tinham relato de histórico conhecido de alergias prévias e 15 (19,0%) apresentavam comorbidades. Oito casos registraram vacinas concomitantes (HPV, DTP, Covid-19, Pfizer pediátrica e Meningo ACWY). O tempo mediano entre a vacinação e o início dos sintomas foi 23 minutos (Q1: 10 e Q3: 40). Não foram detectados conglomerados ou surtos relacionados a lotes e locais de vacinação.

**Conclusão:**

Os casos de anafilaxia, embora graves, tiveram boa evolução sem óbitos. Recomendações incluem a realização de triagem das pessoas a serem vacinadas, comunicação clara sobre indicações e contraindicações, controle de estoque e da cadeia de frio, além de monitoramento para prevenção de Esavi grave. O Ministério da Saúde recomenda a vacinação contra a dengue, uma vez que essa tecnologia em saúde é segura, mas enfatiza a importância de medidas complementares para o controle da doença no Brasil.

